# Understanding the Lived Experience of Fetal and Neonatal Alloimmune Thrombocytopenia: A Qualitative Study

**DOI:** 10.1055/a-2948-6561

**Published:** 2026-09-22

**Authors:** Ellen Elisabeth Johanna de Kreij, Melanie Mesch, Stacy Corke, Maartje Mangelaars, Lisa Shea, Roland Devlieger, R. Katie Morris

**Affiliations:** 1 Patient AuthorLandgraafthe Netherlands; 2 Patient AuthorSan FranciscoCaliforniaUnited States; 3 NAITbabies.orgPenzanceCornwallUnited Kingdom of Great Britain and Northern Ireland; 4 Johnson & JohnsonBredaThe Netherlands; 5 Johnson & JohnsonHorshamPennsylvaniaUnited States; 6 Department of Development and RegenerationKU LeuvenLeuvenBelgium; 7 Department of Obstetrics and GynaecologyUniversity Hospitals LeuvenLeuvenBelgium; 8 Department of Obstetrics, Gynaecology and ReproductionSt-Augustinus Hospital WilrijkWilrijkBelgium; 9 Birmingham Clinical Trials UnitSchool of Health Sciences, College of Medicine and Health, University of BirminghamBirminghamUnited Kingdom of Great Britain and Northern Ireland

**Keywords:** fetal and neonatal alloimmune thrombocytopenia, alloimmune disease of pregnancy, rare disease, patient perspective, lived experience, patient experience

## Abstract

**Objective** Fetal and neonatal alloimmune thrombocytopenia (FNAIT) is a rare alloimmune disease of pregnancy. This study aimed to better understand the disease burden of FNAIT and its impact on families through qualitative research.

**Study Design** This exploratory qualitative study involved thematic analysis of semi-structured virtual interviews and group sessions (Cohort 1: October 2021–November 2024; Cohort 2: April–May 2025) with members of the FNAIT Patient Council from the United States, Canada, the United Kingdom, Australia, and the Netherlands, organized by Johnson & Johnson.

**Results** There were 10 participants (9 female, 1 male) in Cohort 1 and 12 female participants in Cohort 2. Cohort 1 themes included the challenging and inconsistent diagnostic path, responsibility for self-education and self-advocacy, and navigation of unclear care pathways. Cohort 2 themes included the challenging diagnostic path, wider impacts on families and family planning, burdensome treatments, mental health, and opportunities to improve overall experiences.

**Conclusion** These qualitative findings demonstrate the weight of the emotional, logistical, and financial burden placed on families affected by FNAIT. Unmet needs include improved antenatal screening and earlier diagnosis, less burdensome treatment, more psychosocial support, and improved awareness of FNAIT among healthcare providers.

##

**Key Points**

- Fetal and neonatal alloimmune thrombocytopenia (FNAIT) is a rare alloimmune disease of pregnancy
- This qualitative study aimed to better understand the lived experience of parents affected by FNAIT
- Themes included diagnostic challenges, responsibility for self-education and self-advocacy, and navigating unclear care pathways
- Participants also described family impacts, burdensome treatments, mental health, and improvement opportunities
- These findings highlight needs for improved screening, diagnosis, treatment, psychosocial support, and healthcare provider awareness

## Introduction

Fetal and neonatal alloimmune thrombocytopenia (FNAIT) is a rare alloimmune disease of pregnancy; it is estimated to affect approximately 1 in every 1000–2000 pregnancies. 1 2 3 Maternal alloantibodies against fetal human platelet antigen (HPA) cross the placenta via the neonatal Fc receptor, leading to platelet destruction and varying degrees of fetal or neonatal thrombocytopenia. 1 4 HPA-1a incompatibility is the most common cause of FNAIT and is the major cause in 75–80% of FNAIT cases in people of European ancestry. 5 6 Approximately 2% of Caucasian women are HPA-1a-negative, with a lower prevalence in other races. 6 Different antigens (e.g., HPA-4b, HPA-5b, and CD36) may predominate in Asian and African populations. 5 Accordingly, the prevalence and severity of FNAIT may vary among different racial and ethnic groups. Fetal and neonatal intracranial hemorrhage (ICH) caused by FNAIT occurs in approximately 1 in every 10,000 pregnancies, largely in utero, and may result in fetal or neonatal death or severe neurologic damage. 1 3 7

Current antenatal screening for FNAIT focuses on identification of high-risk pregnancies in women who have experienced a previous FNAIT pregnancy, in particular fetal and neonatal ICH. 8 While practice recommendations have been adopted globally for the antenatal management of high-risk pregnancies where ICH has previously occurred, there is currently no international consensus for standard-risk pregnancies (i.e., all non-high-risk pregnancies) where ICH has not happened before; there are also no universal HPA-1a screening programs or approved therapies for the prevention of FNAIT. 1 Although management approaches differ across countries, 8 9 10 they typically involve antenatal, off-label, weekly maternal intravenous immunoglobulin (IVIG) infusion, with or without corticosteroids, 1 8 11 12 along with postnatal IVIG and/or HPA-matched/unmatched platelet transfusion for the newborn. 10 11 Some questions remain around the risk–benefit profiles of these treatments, particularly in standard-risk FNAIT pregnancies with no prior history of ICH. 13 14 15

Little is currently known about the broader burden and impact of FNAIT on parents. One survey study has shown that more than half of FNAIT mothers report psychosocial challenges, 16 and the burden of weekly IVIG treatment and frequent fetal monitoring and surveillance is likely to further exacerbate maternal stress and anxiety.

This study’s goal was to better understand the lived experience of parents affected by FNAIT pregnancies. It examined parents’ experiences in first and subsequent pregnancies and through diagnosis and treatment pathways—including the impact of any medical care received. It also looked at how FNAIT can affect parents in terms of mental and emotional well-being and future family planning. The overall aim of the study was to provide greater understanding of unmet needs in FNAIT and help inform future care and support needs.

## Methods

### Study Design

This was an exploratory, phenomenological, qualitative study designed to identify themes based on the self-described lived experience of parents impacted by an FNAIT pregnancy. The study was conducted in two research cohorts. Cohort 1 comprised individual, semi-structured, virtual interviews and eight group sessions held between October 2021 and November 2024 with members of Johnson & Johnson’s existing FNAIT Patient Council Advisory Board from the United States and Canada. To better expand the global representation beyond Cohort 1, the interviews were also conducted with an additional group of participants (Cohort 2) from Canada, the United Kingdom, Australia, and the Netherlands during April and May 2025.

The study was conducted in accordance with the Declaration of Helsinki and received ethical approval review exemption from the WCG Clinical Institutional Review Board, pursuant to the terms of the U.S. Department of Health and Human Services Policy for Protection of Human Research Subjects (45 C.F.R. §46.104(d)). All participants signed a consent and release form that communicated confidentiality and compliance with the U.S. Health Insurance Portability and Accountability Act, the European General Data Protection Regulation, and the U.K. Data Protection Act. Participants were compensated for their time attending the engagements. The study findings are reported in line with the COnsolidated criteria for REporting Qualitative research guidelines. 17

### Participants

Participants were identified for both cohorts via NAITbabies.org, a U.K.-based charitable incorporated organization run by families with FNAIT, using purposive sampling. They were recruited via email invitation. Eligible participants were adults (aged ≥18 years) with a self-reported diagnosis of FNAIT during a previous pregnancy living in one of the countries listed in the Study Design section. Male participants were eligible if they were a partner of a female participant. A total sample size of 20 to 25 was targeted, in line with standard data saturation approaches in phenomenology. 18

### Procedures

All participants took part in an individual, 1-hour virtual interview. Those in Cohort 1 also attended eight 2-hour virtual focus group sessions. Interviews and group sessions were moderated by a professional research specialist with 5 years’ experience in qualitative healthcare research, including moderation and qualitative analyses (from the Center for Information and Study on Clinical Research Participation [CISCRP]; Boston Massachusetts, United States). The moderator was not previously known to the participants. Interviews and focus group discussions were attended by an additional observer from CISCRP and were moderated in a semi-structured format, including questions and prompts to elicit honest feedback and opinions. A moderator guide of overarching question topics, used for both cohorts, was developed collaboratively by the research personnel from CISCRP in conjunction with the sponsor ( **Supplementary File S1** , available in the online version only).

At the beginning of each interview and focus group, the moderator introduced herself and presented the goals of the research, which were also outlined in the consent documentation. For Cohort 1, the objective was to learn about participants’ lived experience of FNAIT diagnosis, treatment, and support. Interviews for Cohort 2 looked at participants’ diagnostic and treatment experiences, the impacts on family life and family planning, mental health impacts of FNAIT, and participants’ thoughts on how their unmet needs could be improved.

The moderator and observer made field notes during each engagement, and all sessions were audio-recorded and transcribed. Transcripts were anonymized and were not shared with the participants.

### Data Analysis

Data analysis was led by the moderator, using a phenomenological thematic analysis to draw insights from interactions with participants. 19 Following debrief discussions between the moderator and observer, transcripts were reviewed manually and analyzed using a six-step analytical method to identify key themes and patterns. 19 During this process, multiple, independent, qualitatively trained research personnel first reviewed each transcript twice to familiarize themselves with the data and make direct observations. Thematic codes were assigned to certain words or sentences in the transcripts to help organize and reveal patterns across the data in Microsoft Word. Finally, the analyst identified themes and sub-themes from those labels based on a priori knowledge of the condition, using both inductive and deductive reasoning. Annotated transcripts, categorized by key themes, were analyzed using qualitative research analysis software (MAXQDA; VERBI Software, Berlin, Germany).

## Results

### Participant Characteristics

Cohort 1 included 10 participants (9 female, 1 male) with experience of FNAIT ( Table 1 ). Nine were U.S. residents and one was from Canada. The male participant was a partner of one of the female participants. Cohort 2 consisted of 12 female participants, 7 of whom were based in the U.K., 2 in Canada, 2 in Australia, and 1 in the Netherlands ( Table 1 ).

**Table 1:** Participant demographics.

Characteristic	Cohort 1 ( *n* = 10)	Cohort 2 ( *n* = 12)
Sex at birth, *n* (%)		
Female	9 (90.0)	12 (100)
Male	1 (10.0)	0
Age group at the time of study, y, *n* (%)		
20–29	0	1 (8.3)
30–39	4 (40.0)	5 (41.7)
40–49	6 (60.0)	4 (33.3)
50–59	0	2 (16.7)
Race, *n* (%)		
White	8 (80.0)	12 (100)
Preferred not to disclose	2 (20.0)	0
Country of residence, *n* (%)		
The United States	9 (90.0)	0
Canada	1 (10.0)	2 (16.7)
The United Kingdom	0	7 (58.3)
Australia	0	2 (16.7)
The Netherlands	0	1 (8.3)
Highest educational attainment, *n* (%)		
High school	1 (10.0)	0
Some college or vocational training	1 (10.0)	1 (8.3)
Bachelor’s/undergraduate degree	4 (40.0)	7 (58.3)
Master’s/postgraduate degree or higher	4 (40.0)	4 (33.3)

During outreach, no invitees declined to participate in the study. Partway through the study, one participant from Cohort 1 did not respond to requests for further participation; data from this participant’s initial research engagements have been included in the analysis.

Other than two participants in Cohort 1 who opted not to disclose their race, all participants were White. Most participants in both cohorts had completed education beyond high school.

Most participants in each cohort had experienced ≥2 FNAIT-affected pregnancies (Cohort 1: 7 of 9 [77.8%; male partner participant excluded to prevent duplication]; Cohort 2: 11 of 12 [91.7%]; Table 2 ). Two participants each in Cohort 1 had four pregnancies affected by FNAIT. Further, five participants in Cohort 1 and two participants in Cohort 2 had experienced baby loss. Most participants had been treated with IVIG, with or without corticosteroids, during subsequent pregnancies; four participants (two in Cohort 1 and two in Cohort 2) reported having received no treatment for their subsequent pregnancy or having had no subsequent pregnancies.

**Table 2:** Clinical characteristics.

Characteristic	Cohort 1 ( *n* = 9) ^a^	Cohort 2 ( *n* = 12)
Number of FNAIT-affected pregnancies, *n* (%)		
1	2 (22.2)	1 (8.3)
2	5 (55.6)	10 (83.3)
3	0	1 (8.3)
4	2 (22.2)	0
Experienced baby loss, *n* (%)	5 (55.6)	2 (16.7)
Treatment for subsequent pregnancies, *n* (%)		
No treatment or no subsequent pregnancies	2 (22.2)	2 (16.7)
IVIG ^b^	4 (44.4)	5 (41.7)
IVIG + corticosteroid ^b^	4 (44.4)	5 (41.7)

Abbreviations: FNAIT, fetal and neonatal autoimmune thrombocytopenia; IVIG, intravenous immunoglobulin.

^a^ The male participant was the partner of a female participant and is excluded from pregnancy calculations to prevent duplication of data.

^b^ One female participant was treated with both IVIG alone and IVIG + corticosteroid for separate subsequent pregnancies; therefore, the total exceeds *n* = 9.

### Qualitative Analyses: Cohort 1

**Fig. 1:**
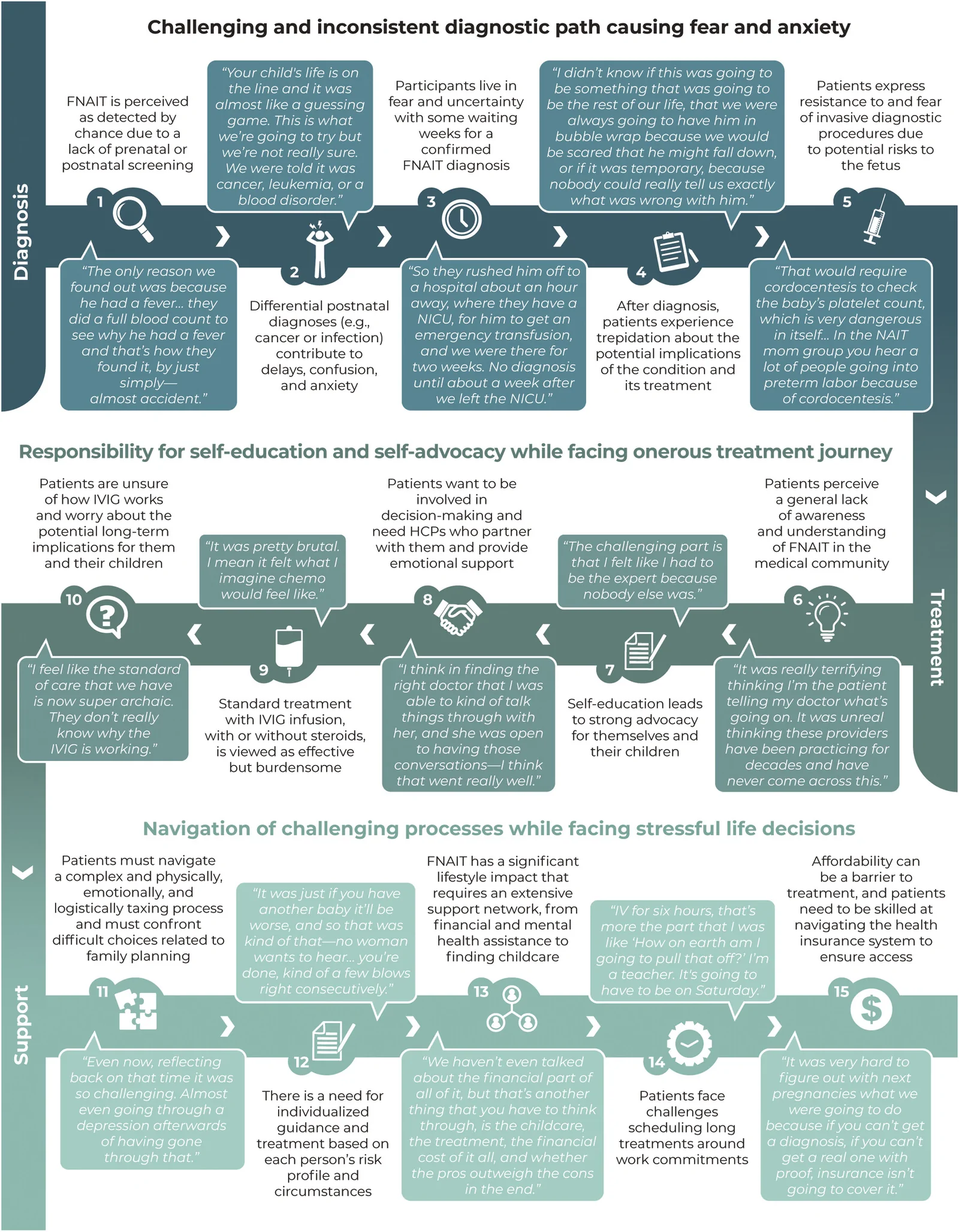
Participant perspectives of diagnosis, treatment, and support needs in fetal and neonatal alloimmune thrombocytopenia (FNAIT; cohort 1; *n* = 10). FNAIT, fetal and neonatal alloimmune thrombocytopenia; HCP, healthcare provider; IV, intravenous; IVIG, intravenous immunoglobulin; NICU, neonatal intensive care unit.

Analyses of participant insights from Cohort 1 highlighted the profound effect of FNAIT. Participants described fear and uncertainty during diagnosis, significant treatment burden, and anxiety around the long-term implications of FNAIT ( Fig. 1 ).

#### Challenging and Inconsistent Diagnostic Path Causing Fear and Anxiety

Participants described diagnostic journeys that were highly inconsistent (Box 1). For some, a diagnosis of FNAIT was given within days after presentation; others faced weeks of delay before receiving a diagnosis, during which time they lived in fear and uncertainty. There was a general perception among the cohort that FNAIT was diagnosed by chance, and there was some resistance to, and fear of, the diagnostic procedures, including universal reluctance to undergo invasive diagnostic procedures such as cordocentesis and amniocentesis due to their associated risks to the fetus. While several participants described how their healthcare provider (HCP) had suggested FNAIT based on their newborn’s symptoms (e.g., fever, rash/petechia, or bruising), others only heard about FNAIT after the findings of unrelated laboratory tests or after their newborn required emergency platelet transfusions. Often, other diagnoses were initially suspected (e.g., cancer or infection), resulting in diagnostic delay, significant confusion, uncertainty, and fear.

*“Your child’s life is on the line and it was almost like a guessing game. This is what we’re going to try but we’re not really sure. We were told it was cancer, leukemia, or a blood disorder. Not sure which one we really wanted it to be if we could have picked and hoped for one.”* Female, U.S.

Participants described ongoing feelings of trepidation during and after diagnosis as they came to terms with the potential implications for their baby—from both the condition and its treatment.

#### Responsibility for Self-education and Self-advocacy while Facing Onerous Treatment Journeys

Participants described how lack of experience and knowledge about FNAIT among the medical community added to their frustration and fueled anxiety and mistrust during their treatment journey. They felt compelled to become experts on the condition and advocate for themselves and their babies. They described how being forced into the roles of educator and advocate was particularly overwhelming given the circumstances they were facing, such as the burdensome treatment protocol.

**Box 1 Clinical perspective on how FNAIT should be diagnosed**

Development of widespread petechiae and/or ecchymoses in a newborn within minutes of delivery with severe thrombocytopenia (platelets < 10 × 10 ^9^ /L) raises strong clinical suspicion of FNAIT. 1 To confirm a suspected diagnosis, laboratory investigations should be undertaken to identify fetal/neonatal and maternal HPA genotype/phenotype (to confirm a feto-maternal incompatibility) and the presence of maternal alloreactive antibodies against the implicated antigen. 1 While fetal platelet counts can be determined by cordocentesis, this invasive procedure is not usually recommended due to associated risks. Unlike hemolytic disease of the fetus and newborn, historically, there have been no approved methods to screen for fetal thrombocytopenia; however, antenatal fetal ultrasound or magnetic resonance imaging can be used to screen for ICH and monitor fetal growth restriction.

*“There was a lack of knowledge about this condition whatsoever that made it extremely frustrating for me, as someone dealing with it, to have to be the one to constantly give information and justify it.”* Female, U.S.

Participants emphasized their need to be involved in their treatment journey and described how they valued HCPs who treated them as partners in the process. All but one of the participants in Cohort 1 had received IVIG infusions with or without steroids during their subsequent pregnancies (Box 2). While they viewed IVIG as generally effective, those who had received it described it as inconvenient, uncomfortable, and exhausting, with significant side effects. Depending on their specific IVIG protocol, participants shared how they spent either a half or full day once or twice a week undergoing infusions throughout many weeks of their pregnancies. Participants likened the exhaustion that accompanied treatment to the effects of flu or chemotherapy. They voiced concerns about their baby’s exposure to pathogens during IVIG therapy and/or transfusions and potential long-term implications of a treatment with a mechanism of action not fully understood by the medical community. 20 These concerns manifested in the need for reassurance through frequent monitoring (e.g., regular ultrasounds) during treatment and emotional support from the healthcare team. Those who received steroids described additional worries regarding the risk of side effects, including gestational diabetes.

**Box 2 Clinical perspective on treatment of high-risk FNAIT**

When an FNAIT pregnancy is known to be high risk (i.e., having experienced ICH in a previous FNAIT pregnancy), maternal treatment typically includes off-label weekly administration of high-dose IVIG in pregnant HPA-1a alloimmunized women. 1 21 Despite limited clinical trial evidence, IVIG treatment is now well established as the standard-of-care treatment in FNAIT 13 ; however, the optimal dose (typically 0.5–2 g/kg/week) and timing of treatment initiation (usually 12–16 weeks’ gestation) remain unclear. 1 21 Corticosteroids are often administered in combination with IVIG in the United States, and occasionally in some European countries; however, optimal dose and timing also vary for this treatment, and questions remain about its use. 1 Neonatal treatment includes platelet transfusion (preferably HPA-matched) to maintain platelet count > 30 × 10 ^9^ /L. 22

#### Navigation of Challenging Process while Facing Stressful Life Circumstances

Participants described their lived experience of FNAIT as a complex and physically, emotionally, and logistically taxing process, involving difficult discussions around family planning. They discussed the choice they had to make between resigning themselves to not having more children or choosing to undergo a treatment that they knew would be challenging. In addition to the physical toll of FNAIT and its treatment, participants described the wider impact of the condition, including experiences of depression, anxiety, and post-traumatic stress. Financial impacts were also important when considering the costs and employment considerations associated with repeat hospital attendance; additionally, IVIG treatments may not always be eligible for reimbursement in some insurance-based healthcare systems. These impacts, alongside managing work commitments and childcare around long infusion schedules, highlighted the importance of having an extensive support network, encompassing mental health assistance and childcare.

Several participants described how they felt disheartened when HCPs tried to dissuade them from getting pregnant and expressed a desire for guidance and treatment tailored to each individual case and risk profile.

*“It’s tough to hear after giving birth that somebody that you know and trust in the medical field is advising you to not have any other kids.”* Female, U.S.

### Qualitative Analyses: Cohort 2

Key themes emerged from qualitative analyses of individual interviews conducted in Cohort 2. Themes included difficult experiences during the diagnosis period, wider impacts on the family and on future family planning, experiences with burdensome treatments, impacts on mental health during the pregnancy and the postpartum period, and opportunities to improve overall experiences.

#### Difficult Experiences during the Diagnosis Period

Most participants in Cohort 2 (9 of 12 [75.0%]) reported that their first-diagnosed FNAIT pregnancy was without complications prior to their postnatal FNAIT diagnosis (Box 3). Of the three participants with a baby who experienced complications of the condition during a first-diagnosed FNAIT pregnancy, one had an induced delivery at 37 weeks following a severe ICH and baby loss, for medical reasons based on physician advice. While 7 of 12 (58.3%) participants received a diagnosis of FNAIT after their first pregnancy, 3 only received a diagnosis after their second pregnancy, and 2 after their third pregnancy. Two participants indicated that their babies had subsequently been diagnosed with cerebral palsy due to ICH—one of whom was additionally diagnosed with epilepsy, autism, and dyslexia.

**Box 3 Clinical perspective on why FNAIT is often diagnosed after birth**

Because there is no universal HPA-1a screening program in pregnancy, most (~60%) cases of FNAIT are not diagnosed until after birth, when suspicion is raised due to either clinically visible bleeding symptoms combined with severe thrombocytopenia or detection of a low platelet count during incidental testing. 6 FNAIT often recurs in subsequent histologically incompatible pregnancies, and secondary prevention is possible if at-risk pregnancies can be identified in women who have previously had an FNAIT pregnancy. 5 The 2019 guidelines from the International Collaboration for Transfusion Medicine recommend that women previously affected by FNAIT (and their female siblings) should be referred to fetal medicine centers and be screened for HPA antibodies to determine the risk of FNAIT in subsequent pregnancies. 22 This would allow for timely intervention and prevention of severe ICH and its long-term sequelae. 6

Most participants in Cohort 2 shared how their baby affected by FNAIT had been born with low birth weight, bruising, jaundice, and/or petechial rash. Some participants reported how physicians had initially attributed these symptoms to infection, low blood sugar (in a mother with type 1 diabetes), hemophilia, or birthmarks. While many of these babies were admitted to the neonatal intensive care unit soon after delivery, mothers whose babies had milder symptoms (or mothers who gave birth at home) described how they had to self-advocate and continue reaching out to their HCPs for help as they felt something was wrong.

At the time of their baby’s diagnosis, participants reported feeling shocked, scared, and overwhelmed.

*“But I just remember holding him and crying because it was so scary. You don’t expect it, especially having two pregnancies before and everything being fine. It was scary. And they’re telling you your newborn might have internal bleeding. That’s actually really scary. And then no one has the answers.”* Female, U.K.

Many described that interactions with their HCPs had been difficult due to a perceived lack of knowledge about FNAIT that led to misinformation. Several participants specifically mentioned instances of poor care and incorrect assumptions.

*“I was told on day two that I couldn’t breastfeed anymore because the antibodies were in my milk, which was a really big thing for me because I breastfed my first two until they were two years old... It wasn’t until I found [NAITbabies.org] and they were like ‘you can breastfeed, what are you talking about?’”* Female, U.K.

Of those participants who described positive postnatal experiences with HCPs, one had given birth in a small hospital and another recalled excellent communication and streamlined coordination of care across maternal and pediatric care teams. In general, however, there was a feeling of poor communication between mother and baby care teams. None of the participants felt adequately educated about FNAIT and its implications for future pregnancies, and the few who were provided with printed educational materials found the information difficult to understand because of the unfamiliar technical and medical terms.

#### Wider Impacts on the Family and on Future Family Planning

While most participants (10 of 12 [83.3%]) went on to have another child following their FNAIT-affected pregnancy, all shared how the condition and their experiences had impacted their family planning in some way. Of the two participants who chose not to have more children, one felt she had been misinformed about treatment availability and subsequently regretted her decision.

*“They gave us a plan that if we would go for a second child, it would be a treated pregnancy… So, with the side effects possible and the travel distance, and the impact on our family, we decided to not have further children. That was a very hard decision.”* Female, the Netherlands

Among those who subsequently had a successfully treated pregnancy, several chose to stop expanding their family any further. Reasons included the burdensome treatment protocol, the potential for side effects, the risk of worsening outcomes for the mother and baby, and the constant anxiety and worry.

*“We did toy with the idea of having one more, but I honestly don’t think I could do it again. The mental strain of wondering if, one, your child is healthy when you’re pregnant with that child or if he’s still alive, constantly worrying, I could never put myself throughout that again, or my family.”* Female, U.K.

#### Experiences with Burdensome and Disheartening Treatments

All participants described how, when they learned they were pregnant again, they had sought out (or were referred to) a specialist, most commonly a maternal fetal medicine doctor (MFM). Most reported seeing their MFM and having regular ultrasounds, initially at least once a month and then more frequently (often every 2 weeks as their pregnancy progressed). Additionally, some participants described seeing specialty midwives and/or having their IVIG therapy overseen by a hematologist. Some chose to travel significant distances to receive care from a specialist familiar with FNAIT.

IVIG infusion therapy had been initiated between 12 and 20 weeks’ gestation for all participants. Without exception, they all commented on the burden of IVIG treatment. They described negative physical side effects, such as headache or migraine, nausea, skin itch, rash, fatigue, and aseptic meningitis, and outlined the difficulties of scheduling the long treatments (6- to 48-hour infusions) around work and childcare. All wished they had received more information about how to manage difficult side effects.

Some participants whose babies had required IVIG and/or platelet transfusions following delivery reported feeling disheartened because of how challenging it had been for them to undergo treatment during the pregnancy in the hope that their child would not require any additional treatment.

#### Impacts on Mental Health during the Pregnancy and the Postpartum Period

Having to learn about a new, rare condition; navigating care and support during pregnancy and while postpartum; and caring for a new baby left many participants overwhelmed and in need of support (Box 4). For those who had a subsequent treated pregnancy, anxiety, stress, fear, and worry about their baby’s (and their own) health began as soon as they discovered they were pregnant. They described how traumatic birth experiences, dealing with the negative side effects of IVIG treatment, and the constant worry about the health of their unborn baby left them feeling anxious and alone. Few participants recalled being offered any mental health resources and support during their treated pregnancy or after giving birth.

**Box 4 Clinical perspective on the impact of stress and anxiety on fetal and maternal outcomes**

In one study, more than half (54%) of 232 mothers with FNAIT pregnancies reported psychological problems that lasted >6 weeks. 16 Most (90%) reported anxiety and many recounted stress, depression, and sleep disturbances. The impact of stress and anxiety on fetal and maternal outcomes in FNAIT is unknown. However, in general, maternal antenatal stress and anxiety are associated with preterm birth and adverse neurodevelopmental outcomes for the baby. 23 24 Social support is a potential clinical target to improve postnatal outcomes. 23

#### Opportunities to Improve Overall Experiences

Reflecting on their personal journeys with FNAIT, participants suggested several ways to improve the experiences of future families impacted by the condition ( Table 3 ). Emphasis was placed on establishing better HPA-1a screening and FNAIT diagnosis, increased HCP training and education, better support for families post-diagnosis, and future research into improved screening, diagnosis, and treatments.

**Table 3:** Participant recommendations to improve the lived experience of those affected by FNAIT (cohort 2; *n* = 12).

Participant recommendation	Illustrative participant perspective
**Better screening and diagnostics for FNAIT**	
Access to consistent preconception and/or antenatal screening Genetic testing, counseling, and guidance on wider family planning decisions	*“I wish there was more of a standardized testing in pregnancy early on. If we’d have had that test at the beginning of our pregnancy or early on in our pregnancy and know that we had that, then that would’ve saved my baby’s life because we could have started treatment, and he would have been OK.”* Female, U.K. *“I heard that [FNAIT] can also affect sisters. I have a sister...she has expressed that she’s worried to have children...because there’s no official testing for [FNAIT] in the U.K. so she could go through a whole pregnancy and possibly lose that child or have a brain bleed or something.”* Female, U.K.
**More accessible and less burdensome treatment**	
More affordable treatments with shorter durations of delivery and fewer side effects compared with IVIG Home-treatment options Logistical and financial support with childcare and transportation due to the frequent and time-consuming nature of treatment and follow-up	*“The treatment is just so expensive and so timely and so emotionally and physically hard. I hope in years to come or months or however long it takes, that something is developed that changes that. Because often we’ve already been through so much and then to have to go through that to have a baby just feels extremely unfair. Although I’m glad we have a treatment, I just wish it could be better.”* Female, U.K. *“I don’t know if it’s possible but maybe like home hospital type—like to be able to have the treatment at home. That would be a lot better, I think.”* Female, Australia
**Increased HCP education and training**	
Better understanding of FNAIT and expertise for all HCPs, including nurses involved in IVIG administration	*“I think the education of the people that are delivering the treatment. Because the midwives, the nurses, they don’t know. So, you feel then out on a limb because […] I just felt like some kind of guinea pig.”* Female, U.K. *“I said what treatment is the option and they said there isn’t, you just have to be monitored, but actually now I know there is treatment.”* Female, U.K.
**Additional support for families**	
Mental health support to deal with the trauma related to the condition and treatment Connection to patient support groups Case studies and success stories Resources to support the spouse/partner	*“I’m actually a mental health social worker, so I actually am really lucky that I know what resources are out there as well. And I knew that I would be impacted by his birth, so I referred myself for mental health support, so I had some therapy for probably about six months, which I found really beneficial for me.”* Female, U.K. *“I found the FNAIT Babies Group on Facebook, which was like a lifesaver. They just knew everything and answered all my questions. It was so difficult hearing that from a doctor who also knows nothing, where you have all these questions and you’re so overwhelmed.”* Female, U.K. *“While I had support, my husband really struggled with it because he wasn’t the mother. Dads seem forgotten sometimes as well. I think by the fact when they came and told us, my husband wasn’t even in the room […], sort of shows they don’t really factor in dads, do they?”* Female, U.K.
**Educational resources**	
Accessible and easy-to-digest information about FNAIT for parents and families Information on IVIG side effects and how to manage them Delivery options	*“I think it’s making sure that any information that’s shared is written in plain language and easy to comprehend.”* Female, U.K. *“Just sort of being signposted to somewhere where this can give you more information or actually have […] somewhere where you can go to sort of learn about [FNAIT] and get more information. Even a website, like an actual website with information would just be a good thing.”* Female, U.K.
**Additional research**	
Impact of FNAIT on learning disabilities in children Long-term impact of IVIG treatment for mothers Genetic studies	*“My oldest son is autistic. My youngest son has ADHD. We’re not sure whether that’s just a family link or due to IVIG therapy.”* Female, U.K. *“I think just awareness overall and I will strongly recommend that my daughter gets tested and even my boys, just to make sure. If they have the weird platelets, then they need to be aware of it, right.”* Female, Canada

Abbreviations: ADHD, attention deficit hyperactivity disorder; FNAIT, fetal and neonatal autoimmune thrombocytopenia; HCP, healthcare provider; IVIG, intravenous immunoglobulin.

## Discussion

This qualitative research provides important insights into the lived experience of parents affected by FNAIT. Study participants described profound impacts, ranging from fear and uncertainty during diagnosis, to significant treatment burden and ongoing anxiety about the long-term implications of the condition and its treatment. Due to limited familiarity with the condition and treatment experience among the medical community, participants felt compelled to become experts on the condition so they could advocate for themselves and their babies. They found navigating the confusing diagnostic process and the burden of IVIG treatment to be physically, emotionally, and logistically taxing. They described feeling overwhelmed, particularly when faced with difficult lifestyle choices related to family planning.

Qualitative insights capturing the lived experience of patients are especially important in rare diseases. Patient insights can help to address knowledge gaps in the face of limited clinical evidence and guidelines, provide meaningful data from smaller sample sizes, and reveal how symptoms impact daily life (i.e., the physical, emotional, psychological, and financial burden). This study examined the lived experience of parents affected by FNAIT. To date, key analyses of patient perspectives in FNAIT currently come from a document analysis of online stories by mothers affected by FNAIT 25 and a preliminary presentation from a landscape assessment that combined a targeted literature review with clinician and patient interviews. 26

A major theme emerging from the current study was the burden participants faced due to screening and diagnostic challenges. Unlike another alloimmune disease of pregnancy, hemolytic disease of the fetus and newborn, which has antenatal screening protocols that enable preventative intervention, there is no universal screening program for FNAIT. In first pregnancies, most diagnoses of FNAIT therefore occur postnatally. 6 Participants in this study reported that FNAIT was often diagnosed through incidental postnatal findings, such as severe thrombocytopenia or bleeding complications. During the diagnostic process, participants often found themselves being counseled through the possibility of multiple differential diagnoses before they received a definitive diagnosis of FNAIT. This iterative process was associated with diagnostic delay and prolonged uncertainty, confounding parents’ confusion, anxiety, and fear. Once participants received a confirmed diagnosis of FNAIT, their worries did not disappear. Instead, they expressed fear and trepidation related to the potential long-term consequences of the disease for their baby. In a survey of 232 mothers, 54% reported psychological problems lasting longer than 6 weeks; 90% reported anxiety; and >50% recounted stress, depression, and sleep disturbances. 16 Along with underscoring the necessity for psychosocial support to reduce uncertainty, these combined findings from the current study and the literature highlight the need to develop screening programs and streamline diagnosis in FNAIT to improve patient experiences (Box 5).

Another major theme identified in the present study was the burdensome responsibility of self-education and self-advocacy. Participants often had to be the expert in conversations with their HCPs, advocating for themselves and their babies. They noted differences in knowledge and poor communication between antenatal and postnatal teams. They also expressed frustration at having to educate their own HCPs while confronting uncertainty and difficult lifestyle choices related to family planning. Similar themes, including lack of awareness about FNAIT and a desire to understand the diagnosis, emerged from an analysis of online stories by 12 North American and European mothers affected by FNAIT, where lack of knowledge and training in diagnosis of FNAIT among the medical community led to increased worry and confusion. 25 Together, these findings highlight the need for targeted HCP education to increase identification and diagnosis of FNAIT and to shift the burden of being the “knowledge bearer” from families to the medical care team. Integrated pathways and formation of specialist care teams involving pediatric hematologists, MFMs, and transfusion medicine specialists is one approach to reducing the burden on individual families trying to navigate care across multiple providers.

Participants also highlighted the need to be skilled at navigating the health insurance system in countries without universal healthcare to ensure access to IVIG. Such global and regional differences in care and reimbursement practices are critical factors to consider as they may add to the administrative and logistical burden for families affected by FNAIT. For some study participants, affordability was a barrier to treatment, as a result of indirect costs (e.g., travel costs, childcare costs, time off work, and costs associated with off-label treatments). These challenges underscore the need for equitable access and support, including patient navigation, financial counseling, and centralized specialist care teams, to reduce the administrative, financial, and emotional burden on families affected by FNAIT.

**Box 5 Clinical perspective on the future landscape of FNAIT**

Many research efforts in FNAIT focus on developing screening programs, immunoprophylaxis, and improved treatment options. To identify HPA-1a alloimmunized pregnancies and allow for better monitoring of FNAIT, efforts are currently under way in the Netherlands to establish a national screening program, 8 and in the United States, a noninvasive prenatal testing panel for fetal platelet antigens was launched in 2026. 27 Clinical trials are ongoing to explore a potential anti-HPA-1a monoclonal antibody for prophylaxis and a potential anti-FcRn monoclonal antibody to limit placental transfer and reduce circulation of maternal alloantibodies. 1 Advancements in the screening, diagnosis, and treatment of FNAIT should also include a focus on HCP and patient education, support programs, and the creation of accessible and trustworthy informational materials to aid informed shared decision-making, such as the resources and support options provided by NAITbabies.org. 28

This study had several limitations. First, some participants had experienced multiple FNAIT pregnancies spanning over several years, introducing potential for recall bias. Individual experiences were also highly variable and are not representative of all experiences with FNAIT. Second, all participants who disclosed their race were White. Limited racial and ethnic diversity in the sample may limit the generalizability of findings to the broader population of families impacted by FNAIT. Further, the participants were recruited through response to outreach; therefore, they may represent a population that is generally more engaged with healthcare and research.

Despite these limitations, the study provides valuable insights into the diagnostic and treatment experiences of parents affected by FNAIT. Strong relationships with FNAIT advocacy groups enabled outreach to 21 participants affected by FNAIT pregnancies—a relatively large sample size given the rarity of the condition. Participants were recruited across five countries from three different continents, adding global representation and a depth of understanding to the findings. Further, analyzing two distinct cohorts of participants allowed for more in-depth interviews in Cohort 2 that built on knowledge gained from Cohort 1 and facilitated the gathering of differing perspectives from a second cohort of participants.

## Conclusion

These qualitative findings based on the lived experience of parents affected by FNAIT indicate the weight of the emotional, logistical, and financial burden placed on families by the lack of screening, diagnostic inconsistency and delay, clinician knowledge gaps, limited communication across medical care settings, and prolonged treatment schedules. These findings highlight unmet needs for improved antenatal FNAIT screening and earlier diagnosis, less burdensome treatment, and more psychosocial support for affected families, as well as increased HCP awareness. Addressing these gaps will require improved HCP education, integrated antenatal–postnatal care pathways, and strong support systems.

## Data Availability

The data-sharing policy of Johnson & Johnson is available at https://innovativemedicine.jnj.com/our-innovation/clinical-trials/transparency. Although these data are not currently publicly available for sharing due to privacy or ethical restrictions, requests for sharing can be sent to the corresponding author and will be evaluated on an individual basis.

## Supplementary Material

Ergänzendes Material Supplementary Material
