# Supplementary material for: Understanding the Lived Experience of Fetal and Neonatal Alloimmune Thrombocytopenia: A Qualitative Study
Source: AJP Rep. 2026 Sep 22;16(3):e185–94. doi: 10.1055/a-2948-6561 (PMC13597138; doi:10.1055/a-2948-6561)
Supplement: Supplementary file 1 [file 10-1055-a-2948-6561_29555807.pdf]

Supplementary File S1

Cohort 1 Moderator Guide and Question Prompts

Prompts were utilized flexibly based on participant responses to prior questions and flow of interview.

1) Please tell us a little about yourself
   - Share a hobby/fun fact
2) To get us started, can you please share how your child was diagnosed with FNAIT?
   - Did diagnosis occur during pregnancy or after birth??
3) When and how did you discover your baby/child had FNAIT?

   If symptomatic:
   - Age of child, discovered after noticing initial symptoms, routine exam?
   - What were your child's initial symptoms?
   - What type of doctor did you first see when you noticed these symptoms? What happened when you saw this doctor?
   - What types of diagnostic tests did you/your child undergo?
   - Were you offered genetic testing? Yourself, child's father?
   - Why did you/ the child's father decline genetic testing?
   - Did the doctor(s) think at first it was FNAIT? If not FNAIT, what did they think was causing symptoms?
   - Were you referred to any other specialists?
   - How long did you wait between your child's initial symptoms and diagnosis of FNAIT?
   - Who ultimately diagnosed your child with Fetal and Neonatal Alloimmune Thrombocytopenia? Type of specialist?
   - How did your doctor tell you about the diagnosis?
   - How was the diagnosis explained to you? Doctor's explanation of possible causes of the condition, doctor's explanation of the way the disease impacts the child's health, etc.?
   - Did your doctor tell you the degree of severity of the condition? Mild, moderate, severe case?
   - How do you feel about the way the diagnosis was communicated to you? Why?
   - Did the doctor say anything that was particularly helpful/unhelpful during the diagnosis conversation?
   - What, if any, questions did you have at the time of diagnosis? Was this doctor/specialist able to answer these questions?
   - What types of support and information, if any, were provided to you upon first hearing the diagnosis?
   - What did you do after learning about this diagnosis? Sought information online, second opinion, etc.?
   - Were any treatment options presented to you? If so, which ones?
   - What were your initial reactions to these treatment options?

AJP Rep | DOI 10.1055/a-2948-6561 | © The Author(s).

- How did you decide on treatment for your child? Second opinion, spouse/family role in treatment decisions?

If not symptomatic:

- How was it discovered that your baby/child had FNAIT?
- What type(s) of doctor(s) did you see along the journey to receiving a Fetal and Neonatal Alloimmune Thrombocytopenia diagnosis?
- Which tests or screening procedures did you undergo? Blood samples, etc.?
- Were you offered genetic testing? Yourself, child's father?
- Why did you/ the child's father decline genetic testing?
- Were you referred to any other specialists?
- Who ultimately diagnosed your child with Fetal and Neonatal Alloimmune Thrombocytopenia? Type of specialist?
- How did your doctor tell you about the diagnosis?
- How was the diagnosis explained to you? Doctor's explanation of possible causes of the condition, doctor's explanation of the way the disease impacts child's health?
- Did your doctor tell you the degree of severity of the condition? mild, moderate, severe case of FNAIT?
- How do you feel about the way the diagnosis was communicated to you? Why?
- Did the doctor say anything that was particularly helpful/unhelpful during the diagnosis conversation?
- What, if any, questions did you have at the time of diagnosis? Was this doctor/specialist able to answer these questions?
- What types of support and information, if any, were provided to you upon first hearing the diagnosis?
- What did you do after learning about this diagnosis? Sought information online, second opinion, etc.?
- Were any treatment options presented to you? If so, which ones?
- What were your initial reactions to these treatment options?
- How did you decide on treatment for your child? Second opinion, spouse/family role in treatment decisions?

If diagnosed during pregnancy:

- When in your pregnancy did this occur?
- What type(s) of doctor(s) did you see along the journey to receiving a Fetal and Neonatal Alloimmune Thrombocytopenia diagnosis? Primary care doctor, OB/GYN, other specialists?
- Which tests or screening procedures did you undergo? Amniocentesis, non invasive cell free testing, any testing that the spouse may have undergone?
- Were you offered genetic testing? Yourself, child's father?
- Why did you/ the child's father decline genetic testing?
- Were you referred to any other specialists?
- Who ultimately diagnosed your baby with Fetal and Neonatal Alloimmune Thrombocytopenia? Type of specialist?
- How did your doctor tell you about the diagnosis?
- How was the diagnosis explained to you? Doctor's explanation of possible causes of the

AJP Rep | DOI 10.1055/a-2948-6561 | © The Author(s).

condition, doctor's explanation of the way the disease impacts child's health?
- How do you feel about the way the diagnosis was communicated to you? Why?
- Did the doctor say anything that was particularly helpful/unhelpful during the diagnosis conversation?
- Did your doctor tell you the degree of severity of the condition? Mild, moderate, severe case of FNAIT?
- What, if any, questions did you have at the time of diagnosis? Was this doctor/specialist able to answer these questions?
- What types of support and information, if any, were provided to you upon first hearing the diagnosis?
- What did you do after learning about this diagnosis? Sought information online, second opinion, etc.?
- Were any treatment options presented to you? If so, which? IVIG, steroids, etc.?
- How were these treatment options explained to you? Questions asked?
- What were your initial reactions/thoughts on these treatment options?
- Did you seek a second opinion and/or search for additional treatment options or information before deciding on a treatment?
- Where did you look for information? Which type of doctors did you visit when seeking a second opinion?
- Did you search online or through patient groups to find more information about your treatment options? Specific names of websites and advocacy groups?
- How did you decide on an initial treatment? Discussions with doctors, family, etc.?
- What factors were most important for you when you were deciding on your treatment? How well you thought the treatment would work, how side effects would affect your daily life, how often you must travel for doctor visits, whether it would travel across the placenta, whether it could affect your baby, etc.?
- What was your treatment plan with these IVIG or steroids? Dose, timing (every week, certain weeks, etc.)?
- Did you experience any side effects or burdens with this treatment plan? Accessing treatment, appointments, timing, etc.?

4) Do you mind sharing some of the thoughts or emotions you had after learning about this diagnosis?
5) Overall, thinking about the journey to diagnosis, what (if anything) do you wish had been done differently?
   - How could the experiences of those whose children are being diagnosed with Fetal and Neonatal Alloimmune Thrombocytopenia be improved?
   - Was there any part of your journey to diagnosis that you think was handled very well? Doctors seen, treatment options, care provided, etc.?
   - Was there any part of your journey to diagnosis that you didn't like, or think wasn't handled very well? Doctors seen, treatment options, care provided, etc.?
6) How many children have you had altogether?
   - Was their child with FNAIT their first or second child, etc.?
7) Did the diagnosis of FNAIT have any impact on your family planning (i.e., decisions around deciding to expand your family etc.)?
   - Have you had any more children?

AJP Rep | DOI 10.1055/a-2948-6561 | © The Author(s).

- Can you please tell me more about your experience with these additional pregnancies?
- What additional steps/precautions did you take, if any?

8) Was any additional monitoring recommended for this subsequent pregnancy(ies)?
   - Specific tests and procedures used to monitor?
9) Were any treatment options presented to you during this pregnancy? If so, which?
   - IVIG, steroids, etc.?
   - How were these treatment options explained to you? Questions asked?
   - What were your initial reactions/thoughts on these treatment options?
   - Did you seek a second opinion and/or search for additional treatment options or information before deciding on a treatment?
   - Where did you look for information? Which type of doctors did you visit when seeking a second opinion?
   - Did you search online or through patient groups to find more information about your treatment options? Specific names of websites and advocacy groups?
   - Did you accept/ move forward with this treatment option?
   - Why did you decline/ decide against this treatment option?
   - How did you decide on an initial treatment? Discussions with doctors, family, etc.?
   - What factors were most important for you when you were deciding on your treatment? How well you thought the treatment would work, how side effects would affect your daily life, how often you must travel for doctor visits, whether it would travel across the placenta, whether it could affect your baby, etc.?
   - What was your treatment plan with these IVIG or steroids? Dose, timing (every week, certain weeks, etc.)?
   - What was your experience with this treatment plan? Burdens like side effects, accessing treatment, appointments, timing, etc.?
   - What was your emotional journey throughout these additional pregnancies?
10) What treatment(s) has your child(ren) received for Fetal and Neonatal Alloimmune Thrombocytopenia since diagnosis?
    - Platelet transfusions, etc.?
    - How was this treatment explained to you? What additional question(s) did you have, if any?
    - What were your initial reactions to/thoughts on the doctor's recommendation to do a platelet transfusion?
    - Did you seek a second opinion and/or search for additional treatment options or information before deciding to move forward with the transfusion? Where? Which type of doctors did you visit when seeking a second opinion?
    - What kinds of discussions did you have with your doctor before deciding to move forward with the transfusion?
    - Were any other family members/caregivers/friends/doctors involved with this decision?
    - Could you please tell us more about your experience with this treatment? What challenges, if any, did you/do you experience with this treatment? Fears, side effects, etc.?
    - Has your child been through any other treatments due to FNAIT?
    - Could you please tell us more about your experiences with this treatment(s)?
11) Outside of a cure, what would the ideal treatment for FNAIT be?

AJP Rep | DOI 10.1055/a-2948-6561 | © The Author(s).

- Symptoms addressing, method of administration, etc.?
- If you could change anything about FNAIT treatments/procedures to make them better, what would you change? More treatment and/or screening options, etc.?

12) Since your child's diagnosis, what type of doctors/health care providers do you/your child regularly see to manage FNAIT?
    - Specialists, and frequency of current visits and level of burden?
    - What, if any, frustrations do/did you have with the care options you were provided with? How could your care have been/be improved?
13) In what ways does FNAIT affect your child's life?
    - Most bothersome symptoms, impact on school schedule/after school activities?
    - In what ways, if any, has FNAIT affected your child's long-term health?
    - How does it affect your life as a caregiver?
    - How has this diagnosis affected others in your family (spouse, additional children if applicable, etc.)
14) How easy or difficult is it for you to care for your child with FNAIT?
    - Difficult aspects, support needed?
    - Do you or any other members of your family have a chronic medical condition? Autoimmune conditions?
    - Which one(s)? What are some challenges associated with managing multiple chronic conditions in your family?
15) Has FNAIT placed a financial burden on your family?
    - Cost of care, treatments, etc.?
    - How much, if at all, of the medical costs did your insurance provider cover?
    - Did you receive any other type of financial assistance outside of insurance coverage? Fundraising, foundations, etc.?
    - How did you find out about this type of aid? Doctors, online, Facebook groups, friends/family, foundations?
16) Do you ever look for information about Fetal and Neonatal Alloimmune Thrombocytopenia? Why or why not?
    - What type of information do you usually seek out? Information about new treatments, background information on the condition?
    - Where do you typically look for this information? Doctor, the Internet, social media (Facebook etc.), patient communities, patient advocacy groups, or online forums/blogs?
    - Has the type of information you search for changed over time? If so, how?
17) How easy or difficult is it to find the information you are interested in about Fetal and Neonatal Alloimmune Thrombocytopenia?
    - Is there any information that you tried to find but couldn't? Kid friendly versions?
    - [IF YES] Where would you prefer to find this information? Online, from their doctor, newspaper, television, social media?
    - In your opinion, what would be the best way to make this information more readily available to patients and their caregivers?
18) In your opinion, what sources of information are most credible or trustworthy? Why?
    - What sources of information, if any, would you not trust or consider credible? Why do you say that?

AJP Rep | DOI 10.1055/a-2948-6561 | © The Author(s).

19) Do you belong to any support groups for Fetal and Neonatal Alloimmune Thrombocytopenia?
    - Which one(s)? How did you become connected?
    - How helpful or not have these support groups been? Why or why not?
    - Do you and your child participate in the support group(s) together? Why or why not?

Cohort 2 Moderator Guide and Question Prompts

Prompts were utilized flexibly based on participant responses to prior questions and flow of interview.

1) Please tell us a little about yourself
    - Share a hobby/fun fact
2) Please tell us a little about your family
    - How many children do you have and what are their ages?
3) How and when was alloimmunization or FNAIT first diagnosed?
    - During pregnancy, after pregnancy? Which pregnancy?
    - How did you discover your alloimmunization diagnosis? When? What was the cause? Did you receive any counseling around future pregnancies?
    - What exactly led to this diagnosis, and what tests/assessments were completed? Labs, symptoms, assessments, etc.?
    - What type of doctor did you first see? Your primary care doctor, specialist/obstetrician, maternal fetal medicine specialist (MFM), neonatology and pediatrics/pediatric hematology?
    - Did this doctor refer you to any other specialists? Type of specialist? OBGYN, MFM, hematology?
    - When did you receive this referral? How long had you seen your original doctor/how many appointments, how many pregnancies?
    - If referred to a specialist, did you continue to receive care/monitoring from your original doctor?
    - How did you feel about switching care during your pregnancy? Comfort level? Did you eventually return to your original doctor? Why or why not?
    - How many other diagnoses, if any, did you receive before the FNAIT diagnosis? Misdiagnosis?
    - What type of doctor ultimately confirmed the diagnosis of FNAIT? Did you seek a second opinion? If so, where?
    - How did they explain this condition? Were you provided with any information or resources about FNAIT? Specific information/resources provided?
    - What questions did you have? Potential treatments, disease progression or complications, future pregnancies, cost of care/treatments, standard of care, etc.? How did you know what questions to ask your healthcare providers? Were there any questions at the time that you were afraid to ask?
    - How did you feel/react when you were given the FNAIT diagnosis? Initial reactions, feelings/emotional reaction, etc.?

AJP Rep | DOI 10.1055/a-2948-6561 | © The Author(s).

- What, if any, emotional and/or mental health support or resources were you provided with? Counselor, Patient Peer Support, Patient Advocacy Group, etc.? What, if any, emotional/mental health support do you feel would have been helpful?

4) What did you do after receiving the FNAIT diagnosis?
   - Notify family, research online, change doctors, etc.?
   - Did you or a loved one look for information on your own? If yes, who searched? What sites, sources?
   - What information were you looking for? Treatment, specialists, supportive resources, etc.?
   - How easy or difficult was it to find information about FNAIT?
   - What information was not available that you wish had been? Potential treatments, disease progression, reputable maternal fetal medicine specialist, social/emotional support, future pregnancies, grief resources, etc.?
   - How would you like to have accessed that information? Conversations with doctors, informational brochures/resources, HDFN foundations, patient support groups, social media?
5) Did the diagnosis of FNAIT have any impact on your family planning (i.e., decisions around deciding to expand your family etc.)?
   - Did you have additional pregnancies affected by FNAIT, changes made to desired family size, etc.?
   - Can you please tell me more about your diagnosis experience with these additional pregnancies? Timing, emotional impact, discussions with providers, etc.?
   - What additional steps/precautions did you take, if any? Earlier monitoring/testing, specialists, earlier treatment, etc.?
6) Were any treatment options presented to you during each of your pregnancy(ies)? If so, which?
   - IVIG, steroids, which pregnancy, clinical trials, etc.?
   - How were these treatment options explained to you? Questions asked?
   - Did you seek a second opinion and/or search for additional treatment options or information before deciding on a treatment?
   - Where did you look for information? Which type of doctors did you visit when seeking a second opinion? Specific sources?
   - Did you accept/move forward with this treatment option? Why? Specify treatment journey for each pregnancy (if multiple)?
   - Why did you decline/ decide against this treatment option?
   - What was your treatment plan with these IVIG or steroids? Dose, timing (every week, certain weeks, etc.)?
   - What was your experience with this treatment plan? Burdens like side effects, accessing treatment, appointments, timing, etc.?
7) Throughout your affected pregnancy(ies), what types of doctors/specialists did you see?
   - MFM/specialist, OBGYN, MFM/specialist + OBGYN, midwife?
   - How regularly did you see a healthcare provider(s)? Frequency of each specialist?
   - In general, how far did you have to travel to receive care and treatment for FNAIT? Distance/travel time for each specialist? How manageable was this?
   - Who did you consider to be your primary FNAIT doctor? MFM, OBGYN, etc.?

AJP Rep | DOI 10.1055/a-2948-6561 | © The Author(s).

- How were you educated on subsequent pregnancies and the risk of FNAIT by your providers? Mentions of increased risk, which specialist?
- Were you given a specialist referral for your subsequent pregnancies, or did you have to arrange for a specialist and conversation about FNAIT on your own? Please elaborate.

8) Did your baby require treatment for FNAIT?
   - Yes, no, platelet transfusions, etc.?
   - How was this treatment explained to you? What additional question(s) did you have, if any?
   - Could you please tell us more about your experience with this treatment? What challenges, if any, did you/do you experience with this treatment? Fears, side effects, etc.?
   - Has your child(ren) been through any other treatments due to FNAIT?
   - Could you please tell us more about your experiences with this treatment(s)?
   - What type of providers were involved in your FNAIT baby's(ies) neonatal care?
   - What, if anything, went well in your baby's(ies) neonatal care? What did not go well? Information given, bedside manner, treatment, breastfeeding, conversations, etc.?
   - How did you feel about the continuity of care transitioning from your pregnancy to neonatal care? Were there any issues? How easy or difficult was the transition? Breastfeeding issues?
   - What, if any, after effects or other complications did your babies/children experience as a result of the FNAIT diagnosis? Please explain.
9) Overall, thinking about your experiences with FNAIT from first being diagnosed to today, what (if anything) do you wish had been done differently?
   - Screening, different stages of the journey: diagnosis, monitoring, treatment administration in utero, delivery, homecare, future family planning?
     In your opinion, how could the experiences of families affected by FNAIT be improved? Earlier screening, improved access to specialized MFMs, better communication/delivery of diagnosis or antibody detection, more emotional support, information given in a way they can understand, better communication between care team, more financial support, improved HCP education, improved access to the up-to-date standards of practice?

AJP Rep | DOI 10.1055/a-2948-6561 | © The Author(s).

COREQ (COnsolidated criteria for REporting Qualitative research) Checklist

A checklist of items that should be included in reports of qualitative research. You must report the page number in your manuscript where you consider each of the items listed in this checklist. If you have not included this information, either revise your manuscript accordingly before submitting or note N/A.

Topic	Item No.	Guide Questions/Description	Reported on Page No.
**Domain 1: Research team and reflexivity**			
Personal characteristics			
Interviewer/facilitator	1	Which author/s conducted the interview or focus group?	7
Credentials	2	What were the researcher's credentials? E.g. PhD, MD	7
Occupation	3	What was their occupation at the time of the study?	7
Gender	4	Was the researcher male or female?	8
Experience and training	5	What experience or training did the researcher have?	7
Relationship with participants			
Relationship established	6	Was a relationship established prior to study commencement?	8
Participant knowledge of the interviewer	7	What did the participants know about the researcher? e.g. personal goals, reasons for doing the research	8
Interviewer characteristics	8	What characteristics were reported about the inter viewer/facilitator? e.g. Bias, assumptions, reasons and interests in the research topic	8
**Domain 2: Study design**			
Theoretical framework			
Methodological orientation and Theory	9	What methodological orientation was stated to underpin the study? e.g. grounded theory, discourse analysis, ethnography, phenomenology, content analysis	6
Participant selection			
Sampling	10	How were participants selected? e.g. purposive, convenience, consecutive, snowball	7
Method of approach	11	How were participants approached? e.g. face-to-face, telephone, mail, email	7
Sample size	12	How many participants were in the study?	9
Non-participation	13	How many people refused to participate or dropped out? Reasons?	9
Setting			
Setting of data collection	14	Where was the data collected? e.g. home, clinic, workplace	7
Presence of non-participants	15	Was anyone else present besides the participants and researchers?	8
Description of sample	16	What are the important characteristics of the sample? e.g. demographic data, date	9
Data collection			
Interview guide	17	Were questions, prompts, guides provided by the authors? Was it pilot tested?	8
Repeat interviews	18	Were repeat inter views carried out? If yes, how many?	7
Audio/visual recording	19	Did the research use audio or visual recording to collect the data?	8
Field notes	20	Were field notes made during and/or after the inter view or focus group?	8
Duration	21	What was the duration of the inter views or focus group?	7
Data saturation	22	Was data saturation discussed?	7
Transcripts returned	23	Were transcripts returned to participants for comment and/or	8

Topic	Item No.	Guide Questions/Description	Reported on Page No.
		correction?	
**Domain 3: analysis and findings**			
Data analysis			
Number of data coders	24	How many data coders coded the data?	8
Description of the coding tree	25	Did authors provide a description of the coding tree?	Throughout
Derivation of themes	26	Were themes identified in advance or derived from the data?	9
Software	27	What software, if applicable, was used to manage the data?	9
Participant checking	28	Did participants provide feedback on the findings?	NA
Reporting			
Quotations presented	29	Were participant quotations presented to illustrate the themes/findings? Was each quotation identified? e.g. participant number	Throughout
Data and findings consistent	30	Was there consistency between the data presented and the findings?	Throughout
Clarity of major themes	31	Were major themes clearly presented in the findings?	Throughout
Clarity of minor themes	32	Is there a description of diverse cases or discussion of minor themes?	NA

Developed from: Tong A, Sainsbury P, Craig J. Consolidated criteria for reporting qualitative research (COREQ): a 32-item checklist for interviews and focus groups. *International Journal for Quality in Health Care*. 2007. Volume 19, Number 6: pp. 349 – 357

Once you have completed this checklist, please save a copy and upload it as part of your submission. DO NOT include this checklist as part of the main manuscript document. It must be uploaded as a separate file.
